# Bicornuate Bicollis Uterus with Obstruction of the Lower Uterine Segment and Cervical Prolapse Complicating Pregnancy

**DOI:** 10.1155/2018/8910976

**Published:** 2018-08-13

**Authors:** Kristen Stearns, Antoun Al Khabbaz

**Affiliations:** ^1^Medical College of Wisconsin and Affiliated Hospitals, Department of Obstetrics and Gynecology, 9200 W. Wisconsin Ave, Milwaukee, WI 53226, USA; ^2^University of Illinois College of Medicine-Rockford, Department of Obstetrics and Gynecology, 1601 Parkview Ave, Rockford, IL 61101, USA

## Abstract

Congenital Mullerian duct anomalies are conditions involving the female genital tract. Cases of complex Mullerian duct anomalies with involvement of the renal system are rare. Occasionally, these cases can be associated with obstetrical complications. Cervical prolapse infrequently complicates pregnancy, and an association between uterine malformations and cervical prolapse has not been cited in the literature. We describe the case of a primigravid patient at 38 weeks of gestation noted to have cervical prolapse during evaluation for preeclampsia and labor induction. Obstetrical ultrasound at presentation to the labor and delivery suite revealed a high suspicion for a bicornuate uterus. The patient was delivered by cesarean section due to obstruction of the lower uterine segment of the gravid uterus. Further evaluation post-partum revealed a bicornuate bicolis uterus and renal agenesis. Pregnancies in patients with bicornuate bicollis uterus can be complicated by obstruction of the gravid uterus, resulting in cervical prolapse and necessitating cesarean section.

## 1. Introduction

Congenital Mullerian duct anomalies are conditions involving the female genital tract. They involve abnormalities of the fallopian tubes, uterus, cervix, and/or upper vagina. The etiology of Mullerian duct anomalies is multifactorial. These abnormalities may result from agenesis or failed fusion of the paramesonephric ducts or from failed resorption of the uterine septum in utero. It is estimated that the incidence of various congenital uterine anomalies is between 0.5% and 5.0% [1]. Bicornuate uterus represents approximately one-fourth of such anomalies, whereas didelphic or “double uterus” is among the least common and represents only 8% of these anomalies [2]. Mullerian duct anomalies have been found to be associated with infertility, early pregnancy loss, preterm labor and delivery, and fetal malpresentation [3]. Other studies have found an association between congenital Mullerian duct anomalies and an increased incidence of renal and urinary tract abnormalities, often leading to more complex cases [4].

The etiology of cervical prolapse is also multifactorial and usually occurs secondary to weakening of the supportive ligaments of the uterus. Cervical prolapse occurs rarely in pregnancy and complicates between 1 in 10,000 and 1 in 15,000 pregnancies [5]. Additional factors have been cited as contributors to this phenomenon including multiparity, increased intra-abdominal pressure, genetic predispositions, collagen abnormalities, and history of pelvic floor surgery. Cervical prolapse can result in vascular congestion of the cervix, cervical edema, cervical insufficiency, and dystocia. Studies have also found an increased risk of spontaneous abortion in patients with cervical prolapse [6].

In this case report, we describe the presentation of a nulliparous patient with cervical prolapse, bicornuate bicollis uterus, and obstruction of the lower uterine segment of the gravid uterus by the nongravid uterus.

## 2. Presentation of Case

A 17-year-old gravida 1 para 0 patient at 38 weeks of gestation was admitted to the labor and delivery suite for labor induction secondary to diagnosis of preeclampsia with severe features (hypertension, proteinuria, and a creatinine level of 1.2 mg/dL). The patient had an uncomplicated course of pregnancy prior to this diagnosis. The patient initiated prenatal care at 16 weeks of gestation and had a normal baseline pelvic examination. The fetal anatomic survey at 20 weeks of gestation was also normal.

**Figure 1:**
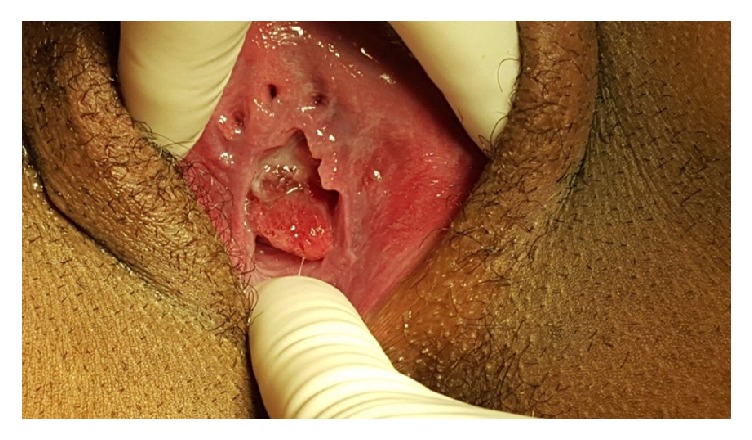
Cervical prolapse reaching the level of the introitus.

Pelvic examination on admission to the labor suite revealed a moderate to severe cervical prolapse with the cervix noted at the introitus (Figure 1). Digital examination revealed a closed cervix, and a posterior mass was suspected in the lower uterine segment. Transvaginal ultrasound demonstrated a mass posterior to the cervix, resulting in displacement of the gravid uterus markedly anteriorly. On ultrasound, the mass appeared to be uterine in origin with normal appearing myometrium and decidualized endometrium. Initial findings were suggestive of a uterine malformation with obstruction of the lower uterine segment of the gravid and anterior left-sided uterus by its nongravid, right-sided, and posterior counterpart.

**Figure 2:**
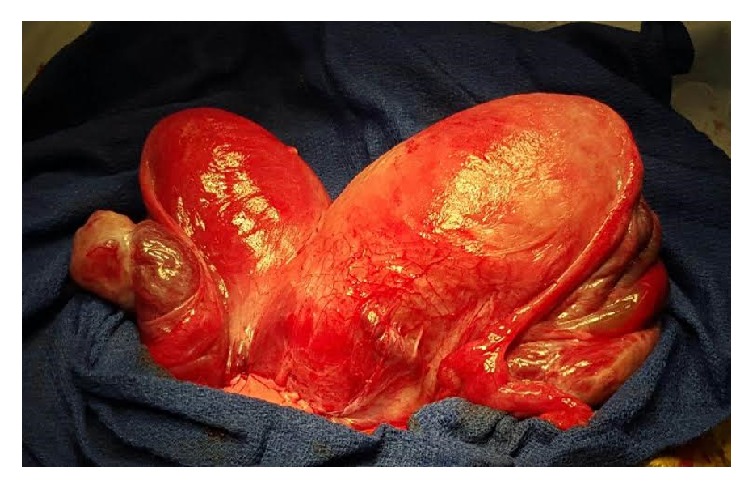
Intraoperative finding of bicornuate uterus (right hemiuterus on the left side of image, left hemiuterus on right side of image).

**Figure 3:**
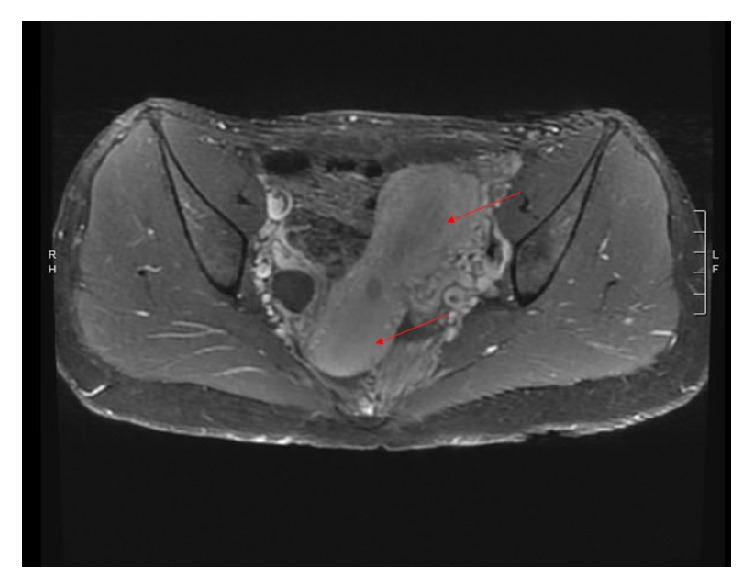
MRI demonstrating bicornuate uterus.

**Figure 4:**
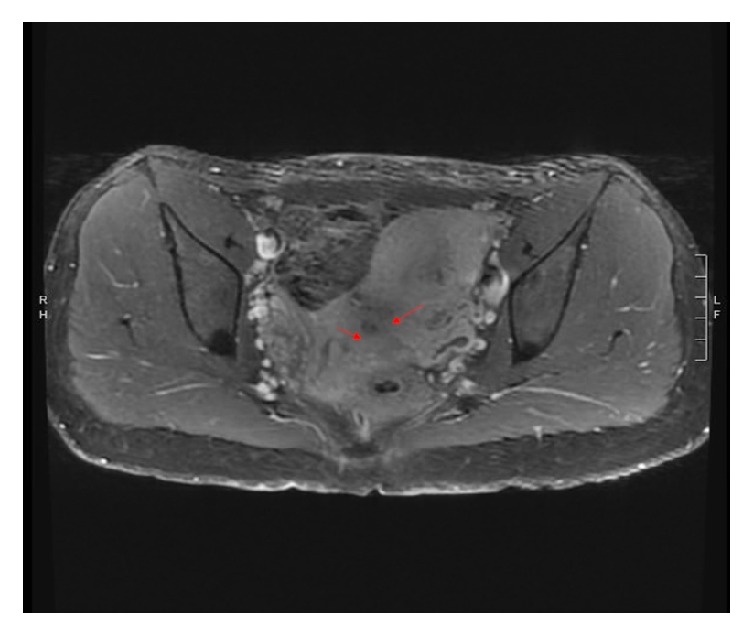
MRI demonstrating bicollis uterus.

The patient was counseled about the need for delivery due to preeclampsia. Complete obstruction of the lower uterine segment of the gravid uterus prompted recommendation for primary cesarean section. A primary low-segment transverse cesarean section via Pfannenstiel skin incision was performed after obtaining patient's informed consent. The patient delivered a live female newborn from a vertex presentation with a birthweight of 2840 grams and Apgar scores of 8 and 9 at one and five minutes, respectively. Intraoperative findings included apparently noncommunicating uteri and normal fallopian tubes and ovaries (Figure 2). The patient received magnesium sulfate prophylaxis for seizures for 24 hours postpartum. During admission, renal ultrasound revealed an absent left kidney with compensatory hypertrophy of the right kidney. Her postpartum course was uncomplicated. Creatinine level normalized postpartum. The patient was discharged home on postpartum day 3. At her 6-week postpartum check-up, speculum examination revealed two cervixes, with the right cervix notably smaller and more superior than the left. There was no evidence of a vaginal septum. Cervical prolapse was noted to have resolved. At 8 weeks postpartum, pelvic MRI demonstrated bicornuate uterus (Figure 3) with cervical bicollis (Figure 4). There was no evidence of communication between the two uterine horns on MRI. Postpartum hysterosalpingogram was not performed secondary to patient loss to follow-up.

## 3. Discussion

**Figure 5:**
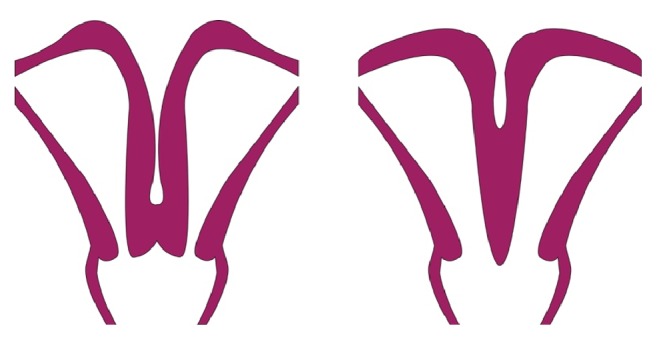
Didelphic uterus (left), bicornuate bicollis uterus (right), courtesy of Kyle Koniewicz.

We report the case of a primigravid patient with bicornuate bicollis uterine anatomy, cervical prolapse, preeclampsia, and unilateral renal agenesis who was delivered with cesarean section due to obstruction of the lower uterine segment of the gravid uterus. Bicornuate uterus is a common Mullerian duct anomaly and can be accompanied with a single cervix (unicollis) or a double cervix (bicollis) depending on the extent of the duplication. Differentiating bicornuate bicollis uterus from didelphic uterus can be challenging, as the anatomy of these anomalies is similar. The key difference between these anomalies is that a didelphic uterus has two widely spaced and completely separate uterine cavities. By comparison, bicornuate anatomy demonstrates some degree of fusion between the two uterine horns, although the septum can extend to the level of the cervix to yield two cervices in some cases (Figure 5). Pelvic MRI is the modality of choice for differentiating the two aforementioned abnormalities.

This case highlights numerous points of discussion including the relationship between bicornuate uterus and cervical prolapse. In this patient's case, the lower uterine segment of the left-sided, gravid uterus was obstructed by the right-sided, nongravid uterus. The cervix of the gravid uterus was displaced downward by its nongravid counterpart, resulting in cervical prolapse. Neither bicornuate bicollis uterus or cervical prolapse is an indication for cesarean section delivery in isolation, and many patients with these conditions are able to progress and deliver vaginally [7]. However, in this case, cesarean section was indicated due to obstruction of the lower uterine segment of the gravid uterus. Bicornuate bicollis uterus and cervical prolapse are relatively rare phenomena. After an extensive literature search, we could not find a case describing the combined presentation of these two conditions.

This case highlights the relationship between bicornuate uterus and preeclampsia. Mullerian duct anomalies are known to be associated with renal and urinary tract abnormalities. Studies have reported that renal anomalies are found in 20-30% of patients with Mullerian duct anomalies, and these cases represent complex mesonephric anomalies stemming from abnormal development of both renal and reproductive anatomy in utero [4]. In this case, further workup postpartum revealed left renal agenesis. The absence of the left kidney probably contributed to the development of this patient's preeclampsia. Heinonen retrospectively studied the possible connection between gestational hypertensive disease and unilateral renal agenesis in women with Mullerian duct anomalies. He concluded that women with uterine anomalies and unilateral renal agenesis have greater than three times the risk for development of preeclampsia than women with normal renal anatomy. This is thought to be a consequence of the increased burden on the solitary kidney due to functional renal changes during pregnancy.

Abnormal uterine anatomy has been well-documented and studied, and complex distal mesonephric congenital anomalies including cases of unilateral renal agenesis and ipsilateral cervicovaginal atresia or an ipsilateral blind hemivagina have been described [8]. Similarly, there have been cases with communicating bicornuate bicollis uterine anatomy associated with atretic blind hemivagina and ipsilateral renal agenesis [8]. Thus, in patients presenting with bicornuate uterine anatomy and unilateral renal agenesis, it is reasonable to suspect anomalies of this nature. This patient did not have evidence of any of the aforementioned cervical and/or vaginal findings. Rather, the patient had apparently noncommunicating uterine horns with respective cervices; a true bicornuate bicollis anatomy with unilateral renal agenesis. This anatomy has not been documented in the literature and represents a very rare anomaly. It is possible that a communication between the uterine horns existed in this patient. It would have been difficult to diagnose at the time of cesarean section and it was not seen on subsequent pelvic MRI. Further evaluation with a hysterosalpingogram could have helped determine if a communication existed between the two uterine horns and if the patient had an atretic cervix.

The finding of cervical prolapse in a pregnant patient at term, particularly in a nulligravid patient, should prompt evaluation for a uterine malformation. In this case, the uterine anomaly was diagnosed at term after finding cervical prolapse and a pelvic mass. The diagnosis was missed at the time of the fetal anatomic survey, likely because the nongravid uterus was positioned posterior to its gravid counterpart. All patients with bicornuate uterus should be evaluated for renal agenesis or other renal and urinary tract malformations. In case of such abnormalities or malformations, these patients should be diligently monitored for hypertensive disease of pregnancy.

## Additional Points

*Teaching Points.* (1) Patients presenting with cervical prolapse and obstruction of the lower uterine segment should be evaluated for a uterine malformation. (2) Uterine malformations can be associated with renal agenesis, which can be associated with preeclampsia.
